# Supplementary material for: Are carbon emissions trading and green financial instruments synergistic? -Comprehensive quantitative research based on content analysis
Source: PLoS One. 2024 Mar 7;19(3):e0298601. doi: 10.1371/journal.pone.0298601 (PMC10919601; doi:10.1371/journal.pone.0298601)
Supplement: S1 Data — (ZIP) [file pone.0298601.s001.zip › Data/地级市Z_F工作报告.zip!/╣π╬≈/╬α╓▌╩╨/└┤╫╘╣1⁄2╓┌║┼í╛┬φ┐╦╩2╛▌═°í┐_2013.docx]

各位代表：

现在，我代表市人民政府向大会作政府工作报告，请予审议，并请各位政协委员和列席会议的同志提出意见。

2012年工作回顾

2012年，面对复杂严峻多变的经济形势，我们在自治区党委、政府和市委的正确领导下，紧紧围绕主题主线，牢牢把握稳中求进的工作总基调，以“三年一工程”为抓手，着力稳增长、强产业、固基础、扩城镇、惠民生、促和谐，保持和扩大了经济社会平稳较快发展的良好势头，圆满完成了市十三届人大二次会议确定的各项目标任务，取得了好于预期的发展成果。

——地区生产总值885亿元，增长15%，高出预期2个百分点，增速排全区第2位；

——全社会固定资产投资855亿元，增长35%，高出预期10个百分点，增速排全区第1位；

——财政收入101亿元，增长32.7%，高出预期12.7个百分点，增速排全区第2位；

——规模以上工业增加值482亿元，增长27%，高出预期5个百分点，增速排全区第2位；

——招商引资实际到位资金539.3亿元，增长35.7%，高出预期6个百分点，增速排全区第2位；

——城镇居民人均可支配收入20610元，增长13%，高出预期1个百分点，增速排全区第4位；

——农民人均纯收入6500元，增长15%，高出预期2个百分点，增速排全区第4位。

一年来，我们主要抓了以下几项工作：

着力做大做强工业，工业经济实力大幅提升。工业规模进一步扩大，全部工业总产值、规模以上工业总产值分别达到1562亿元和1390亿元，分别增长32%和35%，工业总量稳居全区第4位。

科技创新成效显著，获得国家科技项目6项、自治区科技项目71项，拥有3家自治区级的产业化研究院、2家自治区级千亿元产业研发中心、5家自治区级工程技术研究中心，建成1个国家级和1个自治区级质检中心。

技改投入不断加大，完成技改投资324亿元。

重点项目加快推进，年产30万吨再生铜冶炼、金海不锈钢一期等工业项目竣工投产，神冠特色食品医药产业园区暨年产7万吨食用蛋白原料项目开工建设。

积极推动“工业上山”进程，成为全区低丘缓坡荒滩等未利用地开发利用试点城市。国家“城市矿产”示范基地建设取得重大进展，年产值突破500亿元，其中再生不锈钢制品产业园区产值达302亿元、再生资源加工园区产值达210亿元。

全市工业园区完成工业总产值1200亿元，占全市工业总量的76.8%。

着力做好“三农”工作，农业基础不断巩固。粮食生产实现“九连增”，主要农产品供应保障有力。

特色农业加快发展，六堡茶标准化茶园面积6万亩，中药材示范基地面积34万亩，砂糖桔种植面积30万亩；“菜篮子”基地1.1万亩，其中品牌蔬菜基地面积7650亩；规模养殖场发展到4498个。

林业产业快速发展，实现总产值202.4亿元。

农业产业化经营步伐加快，市级以上农业产业化重点龙头企业发展到79家，各类农民专业合作社发展到878家。

农田水利基本建设、病险水库除险加固、水电及农网改造等项目完成投资12.78亿元。

农村公路建设完成投资2.39亿元，新增通硬化路行政村87个。完成2.2万座农村改厕任务，新建沼气池9600座。落实财政扶贫资金8684万元，推进179个贫困村整村扶贫开发工作。投入水库移民后期扶持补助资金3138万元，受惠移民达5万人。

着力发展现代服务业，转型发展步伐明显加快。现代商贸物流业加快发展，市商贸物流园区、毅德商贸物流城、中恒国际医药物流产业园、岑溪泽仁现代商贸物流城等项目加快建设，沃尔玛、国美电器等项目建成营业，全市68个商贸流通业项目完成投资72.9亿元。

落实“家电下乡”政策，全市销售家电下乡产品18.6万台，销售金额达4.9亿元。

扎实推进“万村千乡市场”工程，完成新城市场、大东市场升级改造，新建农家店45家。

电子信息产业园区加快建设，手机制造、信息通道和以掌上多媒体为核心的移动互联网产业进入大招商、大建设时期。

全市社会消费品零售总额257亿元，增长15%。

文化旅游产业加快发展，盛意达电子创意文化城、蝶山六堡茶文化产业园等项目开工建设，骑楼城—龙母庙景区和藤县石表山景区通过国家4A级景区复核，中山公园成功创建为国家3A级景区。

蝶山区和岑溪市分别获得自治区级特色文化产业示范区、特色文化产业项目示范市称号。

现代金融服务业发展加快，招商证券、瑞达期货进驻梧州，北部湾银行、柳州银行在梧设立分支机构。

第一期集优债发债工作顺利启动，东泰公司10亿元债券成功发行。

着力加快城镇化进程，城乡统筹发展水平日益提高。区域性枢纽城市加快形成，南广铁路梧州段全线贯通，梧州至贵港、梧州至柳州、岑溪至水汶和环城高速公路等项目进展加快，长洲水利枢纽三线四线船闸和重点港口码头项目顺利推进，西江机场迁建项目选址获中国民用航空局批复。

红岭新区建设加快推进，玫瑰湖整治工程一期、新图书馆、青少年和妇女儿童活动中心等项目完工，18条市政道路贯通，新区面积拓展近10平方公里。

苍海生态新城项目成湖成景，展示中心建成精品。

旧城改造和城市棚户区改造持续推进，“三冲”地质灾害综合整治工程累计投入超过11亿元。

县域城市化步伐明显加快，归义镇、六堡镇列入广西特色名镇建设试点，石桥小城镇建设项目获国家发改委批复。

完成10800户农村危房改造和6个城乡风貌改造示范点整治任务。

着力推进改革开放，发展活力显著增强。扩权强县工作取得实质性进展，53个市直部门下放到县级的行政审批项目达494项。

医药卫生体制改革扎实推进，国家基本药物制度覆盖率达99.1%，基本药物实际销售价格下降15%。

文化体制改革加快推进，组建了市演艺有限责任公司、市县两级新华书店公司。 13459家。对外贸易进一步扩大，进出口总额达11.9亿美元，增长56.1%。

实施“东向”战略取得重大突破，粤桂合作特别试验区上升为两省（区）发展战略，梧肇战略合作24个重点项目和重要事项计划积极推进。

牵头启动梧玉贵区域经济一体化发展规划编制工作。桂东承接产业转移示范区建设取得新进展，承办了国家西部地区承接产业转移工作座谈会。

成功举办第九届梧州国际宝石节、中国茶叶学会年会暨六堡茶博览交易会和广西（梧州）粤剧节。

产业集群招商成效显著，全市共引进外来投资项目402个，投资总额540.50亿元，其中80%以上的项目来自珠三角等东部地区。

民营经济快速发展，全市民营企业达

着力加强生态文明建设，城市竞争优势进一步显现。循环经济发展实现新突破，成为国家再生资源回收利用试点城市、国家第二批餐厨废弃物资源化利用和无害化处理试点城市、国家级可再生资源建筑应用示范试点城市。

“绿满八桂”造林绿化工程深入实施，完成造林绿化27.4万亩，森林覆盖率达75.5%，城镇绿化面积达41.8万平方米，国家园林城市创建工作取得阶段性成果。

加强西江、浔江和桂江等河流治理，取缔了一批违规采砂场。

生态环境保护力度不断加大，重点推进的85个农村环境综合整治工程取得实效，创建了一批生态乡镇和生态村。

城乡清洁工程深化拓展，获自治区第八届市容环境综合整治“南珠杯”竞赛特等奖。

万元地区生产总值能耗降低3.9%，列入自治区年度减排计划的12个减排项目全部完成，集中式饮用水源地水质达标率、空气质量优良率均保持100%。

绿城水都的城市品牌进一步树立，“山清水秀生态美”逐渐成为城市的核心竞争力。

着力保障和改善民生，人民群众得到更多实惠。为民办的12件实事全面完成，实际完成投资40.6亿元。

“安居乐业”工程深入实施，新增城镇就业3.94万人，农村劳动力转移就业6.11万人，城镇登记失业率控制在3.88%； 8.4‰。

新建保障性住房13880套，新增微型企业2387户。实现城乡居民社会养老保险制度全覆盖，实际参保人数达111.12万人，“五险”参保人数累计达124.6万人次。

新农合参合率98.9%、城镇职工医保参保率97%、城镇居民医保参保率100%。为全市65万农户购买农村住房政策性保险。

城乡低保水平稳步提高，在全区率先提高五保供养标准。完成梧州高中、梧州一中、梧州二中的整体搬迁，全市农村中小学更换了21万套新课桌椅，资助7.8万名贫困学生上学。

市区建成社区卫生服务中心7个。文化惠民工程深入推进，“百团千场”文艺演出“五进”活动和“璀璨广场”群众文化活动蓬勃开展，市博物馆、图书馆和各文化馆（站）均实现免费开放，建成35个村级公共服务中心、223个农家书屋，广播电视村村通工程全面完成。

群众体育活动深入开展，竞技体育取得新突破，成功举办第十一届市运会。

人口和计划生育工作水平进一步提高，低生育水平持续稳定，人口自然增长率控制在

着力创建平安梧州，社会大局保持和谐稳定。社会管理创新工作再创佳绩，“安全宝护营”公益项目荣获全国“社会管理创新最佳案例”奖，维护妇女儿童权益的经验做法在全区推广。

信访维稳工作扎实有效，党的十八大和全区“两会”期间我市实现赴邕进京“零上访”目标，社会稳定风险评估工作机制得到了中央和自治区的高度肯定。

加大社区基层管理力度，推行网格化服务管理，实现社区服务管理“全覆盖”。

突发公共事件应急处置总体预案和专项预案不断完善，应急处置能力得到提升。

“安全生产年”活动取得显著成效，安全生产事故起数、死亡人数分别下降了3.88%、6.41%，安全生产履职综合考评排名全区第一。

食品药品监管工作进一步加强，有效保障了人民群众饮食用药安全。

复合型警务机制改革和农村群防群治工作扎实推进，社会治安动态管控能力明显提升，连续7年获“广西平安市”称号。

在推进经济社会发展的过程中，我们积极转变政府职能，切实加强民主法治建设。自觉接受人大法律监督、工作监督和政协民主监督，认真办理人大代表议案、建议和政协提案，办结率100%。进一步深化行政管理体制和行政审批制度改革，市政务中心申请件达10.2万件，办结同意率99.9%。电子政务覆盖市、县两级，政务公开和政府信息公开工作逐步走向制度化、规范化，政府工作效率和透明度不断提高。坚持从严治政，加强反腐倡廉建设，规范权力运行，强化行政监察和审计监督，惩治和预防腐败体系不断完善，政风行风建设取得新成效。

国防动员、后备力量建设和人防工作继续加强。驻梧部队在维护社会稳定、支持地方建设等方面发挥了积极作用。中直、区直驻梧各单位为梧州经济社会发展做出了突出贡献。编制、法制、民族、宗教、统计、国资、物价、外事、侨务、对台、供销、地震、粮食、农机、移民、住房公积金等工作取得了新的成绩，工会、共青团、妇女儿童、残联、老龄、科协、社会科学、慈善、档案、地方志等工作进一步加强。

回顾2012年工作，我们深感成绩来之不易，在严峻复杂的经济形势面前，全市人民迎难而上，奋力拼搏，在攻坚克难中保持稳定增长，在苦干实干中推进转型发展。这些成绩的取得，是自治区党委、政府和市委总揽全局、正确领导的结果，是市人大、市政协有效监督、大力支持的结果，是全市广大干部群众真抓实干、艰苦奋斗的结果，是社会各界凝心聚力、和衷共济的结果。在此，我代表市人民政府，向全市人民，向各位人大代表、政协委员，向所有关心、支持和参与梧州建设与发展的同志们、朋友们，表示崇高的敬意和衷心的感谢！

在总结成绩的同时，我们清醒地看到政府工作中还存在不少矛盾和问题：经济发展外部压力持续加大，土地、资金等生产要素和环境资源约束日益趋紧；产业结构有待进一步优化，工业主导产业相对单一的问题仍然突出；生产性商贸物流刚起步，还不能完全适应产业发展需要；社会事业发展相对滞后，民生改善离群众的要求还有差距；政府行政效能有待进一步提高，发展环境有待进一步改善等。对此，我们将采取积极有效措施，切实加以解决。

2013年工作主要目标

2013年，是全面贯彻落实党的十八大精神的开局之年，是我市“五年规划三年完成”的决战之年，也是为全面建成小康社会奠定基础的重要一年。当前，国际经济形势依然错综复杂、充满变数，国内经济发展仍面临不少风险和挑战，不平衡、不协调、不可持续问题依然突出。与此同时，我们更要看到，党的十八大描绘了全面建成小康社会的宏伟蓝图，为我市科学赶超、开放崛起指明了方向；自治区党委、政府高度重视、大力支持我市加快发展，特别是粤桂合作特别试验区上升为两省区发展战略，为我市推动经济持续较快发展提供了更广阔的空间和新的契机；我市正处于工业化、城镇化的快速发展期，经济总量不断扩大，竞争实力日益增强，具备了上水平、上台阶的良好基础；更为重要的是，全市上下人心思进，人民群众对美好生活的新期待增添了我们奋力前行的动力，为我们干事创业营造了浓厚氛围。只要我们坚定信心，抢抓机遇，乘势而上，顽强拼搏，就一定能再创新局面，再铸新辉煌。

根据市委的决策部署，今年政府工作的总体要求是：全面贯彻落实党的十八大精神，以邓小平理论、“三个代表”重要思想、科学发展观为指导，围绕主题主线，坚持稳中求进，以提高经济增长质量和效益为中心，深入实施“东向”战略，走好经济发展“三步棋”，唱响城市建设“三部曲”，以“三年一工程”为抓手，更加突出经济结构战略性调整，更加突出强化改革创新，更加突出保障和改善民生，更加突出维护社会和谐稳定，同步推进新型工业化、信息化、城镇化和农业现代化，保持和扩大经济社会发展的良好势头，加快推进富民强梧新跨越，加快建成西江上游经济带的龙头，为全面建成小康社会奠定坚实基础。

全年经济社会发展的主要预期目标是：地区生产总值增长12%，力争15%；全社会固定资产投资增长20%，力争25%；财政收入增长15%，力争20%；规模以上工业增加值增长22%；

社会消费品零售总额增长16%；城镇居民人均可支配收入增长13%；农民人均纯收入增长14%；

城镇登记失业率控制在4.5%以内；完成自治区下达的节能减排任务；人口自然增长率控制在10‰以内；居民消费价格总水平涨幅控制在4%左右。

2013年主要工作安排

完成今年的目标任务，必须突出抓好以下几项工作：

一、加快产业转型升级，为全面建成小康夯实新基础

加快推进新型工业化。坚持“雁行阵势”梯次布局产业发展，加快培育千亿元、百亿元产业，力争年内再生资源、再生不锈钢制品、电子信息产业产值均达到300亿元以上，陶瓷、建材、机械制造产业产值均达到150亿元以上，食品、医药产业产值均达到50亿元。

加快培育以新一代信息技术、节能环保、新材料为主的战略性新兴产业，重点抓好鑫华通科技园、志达电子科技园等项目建设，不断扩大新兴产业规模。

加快传统产业转型升级，重点抓好神冠1万吨微晶纤维素项目、骆驼华南电池免维护及新能源铅酸蓄电池、鸿图公司2万吨精密压铸及模具生产等100个重点技改项目，力争技改投资完成386亿元。

深入实施“抓大壮小扶微”工程，重点扶持107家骨干企业上规模，力争年产值100亿元以上企业发展到3家、50亿元以上企业5家、30亿元以上企业13家、10亿元以上企业37家，产值超亿元的工业企业达250家以上，规模以上工业企业总数达到450家，新发展微型企业2500家以上。

加大企业上市扶持力度，积极推动奥奇丽公司、金茂钛白粉公司、广西有色再生金属公司、梧州松脂公司等企业上市。

加快推进农业现代化。着力转变农业发展方式，扎实推进西江经济带梧州特色农业示范区建设，提高农业综合效益，提升农业产业化水平。稳定粮食生产，确保粮食播种面积达238万亩、粮食总产量82万吨。

推进六堡茶、砂糖桔、中草药、品牌蔬菜等特色种植业规模化、标准化生产，力争水果种植面积60万亩、茶叶种植面积6.5万亩、中草药种植面积35万亩以上、品牌蔬菜基地面积1万亩以上；新造高脂松10万亩、高产油茶5万亩以上；

加快生猪、三黄鸡、肉兔、鱼、蜂、蚕等特色养殖业规模化发展，力争养殖业占农业总产值比重达43%以上。

积极构建新型农业经营体系，大力扶持农业龙头企业和农民专业合作经济组织发展，培育市级以上重点农业龙头企业15家以上，新增农民专业合作社30家以上。

加快建设长洲防洪堤南北堤、高旺防洪堤等工程，推进农田水利工程建设，对89座病险水库进行除险加固，抓好12个中小河流治理项目建设。

新建农村饮水安全工程150处，解决16万农村人口饮水安全问题。进一步推进新农村建设，突出抓好一批综合示范样板点。

加快发展现代服务业。加快建设西江经济区域服务中心，提升现代服务业在经济发展中的地位。

安排商贸物流产业项目87项，计划完成投资85亿元，加快推进市商贸物流园、毅德商贸物流城、中恒国际医药物流产业园、旺城广场、岑溪泽仁现代商贸物流城等重点项目建设。

加强城市新区农贸市场规划建设，支持各县（市）建设果蔬、鲜活农产品等专业物流市场。

优化城乡商业布局，建设一批美食、购物、休闲等特色商业街，推进“万村千乡市场”工程建设。加快发展现代物流、科技、信息、金融等生产性服务业，全面提升商贸、餐饮、物业管理等生活性服务业，积极培育服务外包、文化创意、会展经济等新兴服务业。

大力发展新型消费和绿色消费，挖掘农村消费潜力，力争全市社会消费品零售总额达298亿元。

围绕打造区域性旅游目的地和游客集散地，着力提升景区品质和等级，加快推进西江南岸休闲度假核心区、游客集散中心和“10＋2”旅游项目建设。筹建粤桂城市旅游联盟，共同开发“一程多站”、优势互补的“广肇梧”精品旅游线路，推动西江区域旅游一体化。力争全市接待国内外游客总量突破1000万人次，旅游总收入达90亿元。

二、强化项目投资工作，为全面建成小康增强新动力

掀起项目建设新高潮。牢牢把握扩大内需这一发展的战略基点，发挥投资对经济增长最直接、最有效的拉动作用。深入开展“三年一工程”活动，安排重点项目996项，总投资3026.5亿元，年度完成投资810.5亿元。其中，续建项目472项，年度计划投资552.4亿元；年内新开工项目443项，投资257.9亿元。

安排前期工作经费1600万元，推进81项重点前期工作项目，力争部分项目尽快具备开工条件。继续大力实施“安居工程”，建成保障性住房10167套（户）。

力争完成全社会固定资产投资1065亿元。

加快园区项目建设。以“五园八区”为项目建设主战场，持续打好园区建设攻坚战，安排园区（产业）项目307项，总投资1212.2亿元，年度计划投资313.9亿元。其中，梧州工业园区抓好电子信息产业园一期、污水处理厂二期、创业基地等50个项目，争取年内完成投资78亿元以上；再生资源加工园区抓好年产30万吨再生铝、年产20万吨光亮铜杆、年产130吨稀贵金属综合回收工程等64个项目，争取年内完成投资59亿元以上；陶瓷产业园区抓好远方陶瓷制品生产、新舵陶瓷生产、宇豪陶瓷生产等21个项目，争取年内完成投资28亿元以上；再生不锈钢制品产业园区抓好永达不锈钢技改、金海100万吨不锈钢管材加工等16个项目，争取年内完成投资27亿元以上。

继续实施“一线工作法”、“协同管理制度”，完善重点项目跟踪服务机制，促进园区建设项目早开工、早竣工、早投产，发展壮大产业园区规模，力争全市工业园区实现工业总产值1470亿元、工业增加值350亿元以上、税收35亿元。

提高要素保障水平。认真做好项目前期工作，抓好土地、拆迁、资金、水电等保障要素落实，为项目快速推进创造条件。

充分利用“低丘缓坡”政策，优化利用土地空间布局，优先保障重大工程、重点项目和民生工程的用地。

有效对接国家、自治区发展规划，精心策划一批重大产业项目，争取上级更多的政策支持。加强政银企对接，加大对重点项目、重点企业信贷投放力度。力争新增贷款80亿元，政府融资22亿元。

加强生产要素的组织协调，优先满足重点企业发展需要。

三、推动新型城镇化，为全面建成小康打造新引擎

加快推进交通网络建设。完善“丰字型”铁路网，建成南广铁路梧州段和梧州南站，推进梧州港赤水作业区进港铁路建设，加快柳肇铁路、洛湛铁路电气化、洛湛铁路二线工程前期工作。

完善“一环七射三连线”高速公路网络，确保岑溪至水汶、梧州至贵港高速公路梧州段建成通车，加快梧州至柳州高速公路梧州段、环城高速公路建设，推动梧州至封开高速公路项目前期工作，力争开工建设荔浦至玉林高速公路梧州段。

加快西江黄金水道建设，建成梧州港李家庄码头三期扩建工程、紫金村综合码头一期、中储粮码头，加快长洲水利枢纽三线四线船闸建设，做好赤水圩作业区二期、藤县东胜作业区等项目前期工作，推进西江航道、桂江航道整治工程。

启动梧州西江机场建设。

实施国省道干线公路提级改造，完成西江机场进场公路建设，加快苍梧龙圩至藤县潭东“二改一”工程建设，推进207国道东绕城过境一级公路、长洲水利枢纽至赤水沿江一级公路、蒙山（经夏宜）至金田二级公路、藤县津北至长洲三龙大道二级公路前期工作。

实施农村公路建设攻坚大会战，实现建制村100%通沥青（水泥）路。

加快推进中心城市建设。按照“一江两岸三组团”的发展思路，继续唱响城市建设“三部曲”，提高中心城市的辐射力、影响力。大力推进苍海生态新城建设，重点抓好环城水系、苍海公园、西北岸线绿化景观工程、跨湖桥、半岛核心区等18项工程，力争年内完成投资14亿元以上。

加快红岭新区及拓展区开发建设步伐，重点抓好西江三桥、中央商务区、新加坡印象、玫瑰湖景观综合整治工程二期等35个项目建设，力争年内完成投资22.2亿元。

推动南岸片区开发建设，重点抓好西江四桥、塘源工业区道路、高旺八宝塘片区基础设施等项目，力争年内完成投资6亿元。

扎实推进旧城改造工程，重点抓好“三冲”地质灾害综合整治、新兴二路三路主次干道改造、小街小巷整治工程。

大力发展公共交通，加快停车场、健身休闲场地、应急避难场所等公共配套设施建设。

全力以赴争创国家园林城市，大力开展城市绿道、沿江绿带、社区公园建设，基本达到国家园林城市验收标准。继续推进全国文明城市创建工作。

加快推进县城和小城镇建设。充分发挥城镇的集聚效应，打造一批功能完善、特色鲜明的中小城市，加快新型城镇化步伐。大力推进苍梧龙圩与中心城市同城一体化，进一步扩大岑溪、藤县和蒙山县城城区规模，加强石桥、太平、归义等重点镇、中心镇建设，推进产业、人口和资源向县城和中心镇集聚。

推动梧州工业园区、再生资源加工园区、陶瓷产业园区等产业园区建设，促进产业园区开发与小城镇建设互动发展。

加快城区乡镇改街道办事处、村委会改社区居委会步伐。推动户籍制度改革，逐步实现教育、医疗、社保等基本公共服务覆盖城镇常住人口，有序推进农业转移人口市民化。

坚持城镇基础设施和公共服务设施建设向农村延伸，不断提高城乡统筹发展水平。

深入实施城乡风貌改造和城乡清洁工程。

四、深化拓展改革开放，为全面建成小康增添新活力

全力推进开放合作。深化梧肇战略合作，全面落实粤桂两省区《关于建设粤桂合作特别试验区的指导意见》，完成试验区发展总体规划编制，开展试验区启动区控制性规划编制；开展以路网工程为重点的启动区基础设施建设，加快建设跨省界的大园桥主体工程，力争启动区建设初见成效。

启动梧州—云浮战略合作，择机签署框架协议。

积极推进梧玉贵区域经济一体化，完成规划编制工作。争取自治区开展广西桂东承接产业转移示范区基础设施建设大会战。

进一步加强与港澳地区交流合作，促进区域合作向纵深发展。

认真组织参加中国—东盟博览会、桂台经贸文化合作论坛、泛珠三角区域合作与发展论坛暨经贸洽谈会等重大活动。继续办好梧州国际宝石节。

加快转变对外贸易发展方式，积极开拓新兴市场，力争外贸进出口总额增长20%。

着力抓好招商引资。继续实行市领导联系招商工作队制度及“四个一”招商跟踪服务机制，以再生资源、电子信息、陶瓷、先进装备制造业等产业为招商重点，综合开展产业招商、园区招商、驻点招商、节会招商、商（协）会招商，促进产业集群集聚发展。加大资源整合力度，组建5个重点产业园区招商队和7个产业招商队，主攻珠三角、长三角等地区，大力承接东部产业转移，重点产业园区和重点产业招商队完成招商引资240亿元，力争全市招商引资实际到位资金650亿元。加大已签约项目跟踪落实力度，提高项目的合同履约率、开工竣工率和资金到位率，促进招商引资和承接东部产业转移工作迈上新台阶。。

深化重点领域改革。继续推进非税收入收缴管理改革的扩面工作，全面提高非税收入收缴管理工作水平。做好营业税改征增值税试点准备工作，切实把国家结构性减税政策落实到位。深化财政投资评审改革，严把财政投资评审关。加强政府性债务的清理、监管和风险防范，规范发展投融资平台，健全政府投融资、担保平台的决策、监督、执行机制，增强调控和配置社会资本能力。深化医药卫生体制改革，巩固国家基本药物制度，稳妥推进县级公立医院综合改革试点。加快扩权强县改革。积极推进公共资源交易体制改革。

五、突出保障和改善民生，为全面建成小康提供关键支撑

大力实施双倍增计划。推动农业向“接二（产）连三（产）”延伸，培育农民收入新增长点。

开展农村劳动力转移培训，扶持农民工回乡创业和就业，新增农村劳动力转移就业6.55万人次，力争有劳动能力的低收入农户至少有1人稳定就业。

落实各项强农惠农政策，及时将农作物良种补贴、粮食订单直补、农资综合补贴、农机具购置补贴等资金发放到位。

加大整村推进扶贫开发力度，筹措资金1.7亿元，推进179个贫困村整体脱贫；争取扶贫贴息贷款1.5亿元，扶助10家以上扶贫龙头企业和9万户贫困农户发展生产。

落实企业工资正常增长机制，年内企业职工最低工资标准增长15%以上。

加快推进事业单位实施绩效工资，完善义务教育学校、基层医疗卫生事业单位绩效工资制度。继续实施“乐业”工程，城乡新增就业9.3万人以上，确保“零就业家庭”至少有1人稳定就业。

大力完善社会保障体系。推进城乡居民社会养老保险续保扩面，确保参保率达80%以上。

统筹规划养老设施布局，推行机构养老、居家养老、社区养老，筹建1所大型医疗养老机构、1所综合性老年大学，每个城区分别建设2个社区公共服务中心、居家养老和社区养老服务站。

推动医疗保障制度全覆盖，城镇职工和居民医疗保险、新农合参保率均稳定在97%以上，城镇居民医保政府补助标准提高到280元，新农合人均筹资标准提高到340元。

提高城镇低保水平，低保对象补助标准每人每月增加20元。

完善项目征地搬迁安置制度，妥善解决被征地农民的社会保障问题。

加快发展慈善、社会福利和残疾人事业。

大力实施为民办实事工程。顺应人民群众过上更加幸福美好生活的新期盼，以解决人民群众最关心、最直接、最现实的利益问题为重点，筹措资金40亿元以上，切实办好12件民生工程：（1）社保惠民工程。筹措资金4.55亿元，实施城乡居民社会养老保险、城乡居民最低生活保障、残疾人“阳光家园计划”等项目；新建80个农村五保村。（2）健康惠民工程。筹措资金7.64亿元，实施新型农村合作医疗、地中海贫血防治和免费孕前优生健康检查等项目；对15所乡镇卫生院进行危房改造，抓好2个社区卫生服务中心标准化建设，建成1.1万座农村无害化卫生厕所。（3）教育惠民工程。筹措资金2.08亿元，实施职业培训、扩大农村学前教育资源、农村义务教育学生营养改善计划等项目，对考上大学的贫困生、就读普通高中的贫困学生、就读中职的全日制学生进行资助。（4）强基惠民工程。筹措资金1.17亿元，实施农村饮水安全解困工程、基础设施建设扶贫工程。（5）安居惠民工程。筹措资金12.35亿元，实施城镇公租房、安置房、廉租房和城市棚户区改造等项目，完成6000户农村危房改造。（6）土地整治惠民工程。筹措资金2.55亿元，对42个村实施整村推进土地整理，实施地质灾害治理等项目。（7）农补惠民工程。筹措资金5200万元，实施农机具购置补贴、粮食作物良种补贴等项目。（8）生态惠民工程。筹措资金8696万元，实施“绿满八桂”造林绿化工程、森林生态效益补偿、农村环境连片整治等项目，新建农村沼气池4200座。（9）文化惠民工程。筹措资金3511万元，实施农村公益电影放映、公共文化基础设施场所免费开放等项目，完成72个村级公共服务中心建设。（10）通畅惠民工程。筹措资金5亿元，实施183个建制村道路硬化工程，实现建制村全部通沥青（水泥）路。（11）水库移民惠民工程。筹措资金452.8万元，建成6个水库移民新村。（12）就业创业惠民工程。筹措资金3.35亿元，实施大学生科技创业园、农民创业园等就业创业帮扶工程；发展2500家微型企业，解决13000人就业。

六、推进美丽梧州建设，为全面建成小康保持良好生态

加快循环经济发展。大力发展循环产业、绿色经济，推动与青岛新天地集团、湖北骆驼华南集团等大企业的合作，加快“城市矿产”示范基地建设。加快推进梧州陶瓷产业园区、梧州高新技术产业开发区、塘源化工园区等园区生态循环化改造，着力培育一批循环经济示范企业。调整优化再生资源回收网点布局，加快完善区域性再生资源回收利用体系。引导农业产业结构向无害化方向调整，建设绿色农业生态循环系统。加大工业历史和循环经济理念宣传教育力度，筹建市工业博物馆、市循环经济体验馆。

强力推进节能减排。加强重点行业及重点耗能企业能源消费动态监管，在不锈钢、有色金属、化工、建材等行业企业全面推行清洁生产。推进建筑节能和公共机构节能，继续淘汰落后产能。深入开展整治违法排污企业保障群众健康环保专项行动，推进重点企业污染减排，确保污染物稳定达标排放，力争重点减排项目完成“十二五”规划目标的80%以上。加快城镇污水垃圾集中处理设施建设。推进县域生活垃圾“村收镇运县处理”和生活垃圾分类收集试点工作。

重视生态系统建设。启动环境功能区划，划定生态保护红线。

深入实施“绿满八桂”造林绿化工程，重点推进城市绿道、城市公园、江河沿岸和高速公路、铁路两旁绿化景观建设，力争植树造林24万亩。

大力促进资源节约，强化矿产资源管理，严守耕地保护红线。

实施重大生态修复工程，重点抓好天然林、生态公益林保护和退耕还林、沿江防护林和水生动物养护工程建设。推进水土流失综合治理。

加强城乡水环境和空气质量监管，抓好城市水源地和重点城镇备用水源工程建设规划，加大乡镇集中式饮用水水源地保护力度。

开展国家环保模范城市创建前期工作，推进生态市县、生态乡村等绿色环保系列创建活动。

七、发展社会各项事业，为全面建成小康营造和谐环境

加强和创新社会管理。完善社区（村）管理体制和服务网络，提升服务管理功能。加强社会组织的培育和管理，更好地发挥群众性组织的自我管理、自我服务作用。

推行社会稳定风险评估，健全矛盾纠纷专业调处机制，加强社会矛盾隐患排查，从源头上预防和化解社会矛盾。

深入开展领导干部接访下访活动，畅通民意诉求渠道。

加强网络信息管理，营造良好网络环境。

深化“平安梧州”建设，加强社区矫正人员等特殊人群管理服务，构建社会治安网格化防控体系，严厉打击各类违法犯罪活动。

深入开展“安全生产年”活动，不断强化公共安全体系和企业安全生产基础建设，全面落实安全生产责任，有效防范和坚决遏制重特大安全事故发生。

加强应急体系建设，进一步提升防灾减灾能力。

加强食品药品安全监管，积极实施食品安全行动计划，确保人民群众饮食用药安全。

实施创新驱动发展战略。加强科技创新载体建设，筹建西江经济带梧州区域科技服务中心，加快推进梧州高新区孵化器建设，支持市科技创业服务中心创建国家级孵化器。实施企业创新能力提升工程，支持神冠集团创建国家级企业技术中心，建设5家市级工程技术研究中心、5家市级创新型（试点）企业。深入实施知识产权战略，完成发明专利申请量360件。组织5家制造业信息化示范企业开展制造业信息化改造，创建自治区制造业信息化示范城市。实施30项重点科技攻关项目，加快高新技术成果引进与转化，扶持高新技术产业发展。

努力办人民满意的教育。优化教育资源布局，抓好市十四中迁建、二中新校区、红岭新区小学等项目建设。继续推进中小学校安工程和薄弱学校改造工程，改善城乡办学条件。

深入实施学前教育三年行动计划，切实推进义务教育标准化建设，推动义务教育公平均衡发展。

稳步扩大高中办学规模，打造特色品牌学校。深化五年职业教育攻坚，筹建国家职业教育改革与发展试验区。

支持梧州学院、梧州职业学院建设。

加强教师队伍建设，继续实施教学改革年活动，进一步提高教育教学质量。

建立健全学校、家庭、社会沟通联动机制，营造全社会关心、支持教育的良好氛围。

加快文化体育事业发展。深化“璀璨广场”群众文化活动，丰富群众精神文化生活。提升宝石、龙母、粤剧等文化品牌，深入推进六堡茶文化建设，打造西江文化精品。

完善文化产业发展政策环境，加快建设盛意达电子创意文化城等一批文化产业项目。

广泛开展全民健身活动，提高竞技体育水平。

加大体育事业投入，加快市体育场、岑溪市文体中心等项目建设，开工建设市体育中心，全力筹备广西第十三届运动会。

推动其他事业协调发展。完善社区卫生服务网络，规范农村卫生室管理，让城乡居民就近获得基本医疗卫生服务。

完成市人民医院一期搬迁，加快市妇幼保健院扩建项目建设，启动市第二人民医院搬迁工作，进一步落实新农合“一卡通”、“一元钱”看病等便民措施，提高公共卫生服务水平。

加大疾控基本建设投入，提高疾控能力和水平。

深化城乡环境卫生整治，确保顺利通过自治区卫生城复审评估。

坚持计划生育基本国策，提高出生人口素质，推进诚信计生工作和“幸福家园”工程，全面做好人口计生工作。推行流动人口居住证制度管理工作。

进一步做好国防教育和人防工作，加强国防动员和后备力量建设，深化双拥模范城创建成果，巩固和发展军政军民团结。

积极做好民族团结进步模范区创建工作。

继续做好工会、共青团、妇联、外事、侨务、对台、统计、气象、水文、档案、地方志、老龄等工作，促进社会各项事业全面发展。

八、加强政府自身建设，为全面建成小康聚集正能量

新形势、新任务对政府工作提出了新的更高要求，我们要以强化执行力、协同力、创新力为着力点，全面加强作风建设，严格依法行政，深入推进反腐倡廉工作，努力建设人民满意的政府。

　　改进工作作风。全面贯彻落实中央关于改进工作作风、密切联系群众的八项规定和自治区党委、政府的有关实施意见，坚持问政于民、问需于民、问计于民，拜人民为师，向群众学习。坚持尊重民意、珍惜民力，不搞形象工程、政绩工程，到群众最期盼、环境最艰苦、矛盾最尖锐的地方去，多干群众满意、群众乐意的好事实事。坚持实干富民、实干强梧，敢争一流、敢为人先、敢于负责，埋头苦干、真抓实干，力促重大项目、重点工作、重要事项快推进、早见效。坚持高标准、严要求，加强公务员队伍管理，着力整治庸懒散奢等不良风气，改进文风、会风、学风，精简文件、会议、活动，促进公务员队伍以更加过硬的本领，履行职责、服务群众，不断提高政府的公信力。

　　严格依法行政。严格遵守宪法和法律的规定，进一步健全完善各项规章制度，规范行政行为，减少行政审批，形成规范有序、务实高效的政务运行机制。认真落实市人大及其常委会的决议、决定，加强与市政协的工作沟通和协商，充分听取各民主党派、工商联、无党派人士和人民团体的意见，自觉接受人民群众和新闻舆论监督。落实重大决策听证、公示制度，完善重大决策、重大事项风险评估机制。扎实推进政务公开，加大网络问政力度，促进行政权力阳光运行、透明运行。

　　加强反腐倡廉工作。坚持“标本兼治、综合治理、惩防并举、注重预防”方针，全面推进惩治和预防腐败体系建设，做到干部清正、政府清廉、政治清明。全面落实党风廉政建设责任制，贯彻执行领导干部廉洁自律各项规定，深入开展廉政风险防控，不断筑牢廉洁从政防线。强化工程招投标、政府采购、土地出让、资金使用等重点领域监管，加大对重点项目、重要部门的审计监督和行政监察力度，努力从源头上防治腐败。积极推进公款出国出境、公务接待、公车管理制度改革，坚决遏制铺张浪费、奢侈攀比等不正之风。

各位代表，站在新的起点，谋求新的突破，实现新的发展，是全市人民的殷切期盼，是历史赋予的光荣使命。让我们紧密团结在以习近平同志为总书记的党中央周围，在市委的坚强领导下，解放思想，锐意进取，为加快实现富民强梧新跨越、全面建成小康社会而努力奋斗！
